# Conventional NK cells and ILC1 are partially ablated in the livers of Ncr1 ^iCre^Tbx21 ^fl/fl^ mice

**DOI:** 10.12688/wellcomeopenres.11741.2

**Published:** 2017-07-05

**Authors:** Antonia O. Cuff, Victoria Male

**Affiliations:** 1 Institute of Immunity and Transplantation, UCL Medical School, London, NW3 2PF, UK

**Keywords:** NK, liver, ILC1, ILC3, NKp46, Tbet, Ncr1, Tbx21

## Abstract

Mouse liver contains both Eomes-dependent conventional natural killer (cNK) cells and Tbet-dependent liver-resident type I innate lymphoid cells (ILC1). In order to better understand the role of ILC1, we attempted to generate mice that would lack liver ILC1, while retaining cNK, by conditional deletion of Tbet in NKp46+ cells. Here we report that the Ncr1 ^iCre^Tbx21 ^fl/fl^ mouse has a roughly equivalent reduction in both the cNK and ILC1 compartments of the liver, limiting its utility for investigating the relative contributions of these two cell types in disease models. We also describe the phenotype of these mice with respect to NK cells, ILC1 and NKp46 ^+^ ILC3 in the spleen and small intestine lamina propria.

## Introduction

Mouse liver contains two NK cell populations. Conventional NK cells (cNK) are defined by their expression of CD49b (DX5) ^1^, depend on the transcription factor Eomes ^2^, and circulate freely ^1, 3^. The other NK cell population, which expresses CD49a ^1^, depends on the transcription factor Tbet ^2, 3^ and is unable to leave the liver ^1, 3^. There is still some dispute over whether these cells should properly be considered liver-resident NK cells or non-NK type I innate lymphoid cells (ILC1) ^4, 5^: here, we call them “liver ILC1”. In mice, cNK and liver ILC1 are distinct lineages that cannot cross-differentiate ^6^.

The factors involved in the lineage specification of liver ILC1 are already well-understood, but the function of these cells is not yet clear. They produce IFNγ and TNFα, as expected of ILC1, as well as high levels of GM-CSF ^1– 3, 6^, but it is unclear whether the production of these cytokines specifically by liver ILC1, as opposed to by cNK, have any role in health and disease. Tissue-resident ILC1 in some other organs have physiological functions ^7, 8^, so it is also possible that liver ILC1 have some as-yet-undiscovered physiological role.

To answer these questions, we sought to generate mice that would lack liver ILC1 while retaining cNK. Tbet knockout (Tbx21 ^-/-^) mice fulfill these criteria ^2, 3^, but also have alterations in the T cell compartment that would complicate the analysis. Therefore, we crossed Tbx21 ^fl/fl^ onto Ncr1 ^iCre^ mice to produce conditional knockout (Ncr1 ^iCre^ Tbx21 ^fl/fl^) animals, in which Tbet is lost only in cells expressing Ncr1, whose protein product is the NK cell activating receptor NKp46. Here, we report that these mice have a roughly equivalent reduction in both the cNK and ILC1 compartments of the liver, limiting their utility for investigating the relative contributions of these two cell types in disease models. We also note that the loss of Tbet differentially impacts NKp46 ^+^ ILC populations in the spleen, liver and small intestine, suggesting that Ncr1 ^iCre^ Tbx21 ^fl/fl^ mice could have potential as a tool for understanding how and when Tbet is required for NKp46 ^+^ ILC development and trafficking.

## Materials and methods

### Mice

B6(Cg)-Ncr1 ^tm1.1(icre)Viv^/Orl mice ^9^ (RRID MGI:5309017; “Ncr1 ^iCre^”) were acquired from the European Mutant Mouse Archive as frozen embryos and rederived in house. B6.129- *Tbx21 ^tm2Srnr^*/J mice (RRID IMSR_JAX:022741; “Tbx21 ^fl/fl^”) were acquired from the Jackson Laboratory. Ncr1 ^iCre^ mice were crossed onto Tbx21 ^fl/fl^ and the resultant F1 generation was backcrossed onto Tbx21 ^fl/fl^ to produce Ncr1 ^iCre^ Tbx21 ^fl/fl^ conditional knockouts (n = 6) and Ncr1 ^WT^ Tbx21 ^fl/fl^ littermate controls (n = 6).

Although we chose to use floxed-only, rather than iCre-only, littermate controls, we do recognise that iCre transgene expression itself can have an effect on phenotype. Ncr1 ^iCre^ mice are known to have slightly reduced expression of NKp46 on NK cells, although the total number of NK cells (identified as CD3- NKp46+) in these mice is normal ^9^. We confirmed these observations in Ncr1 ^iCre^ mice, compared to Ncr1 ^WT^ littermate controls in our own colony ^10^. Further, we identify NK cells as Lin- NK1.1+, rather than Lin- NKp46+, to avoid potential confounding effects of reduced NKp46 expression.

Mice were sacrificed between 6.5 and 9 weeks of age, using rising carbon dioxide followed by cervical dislocation. Spleen, liver and intestines were dissected out of each of the 12 mice for cell isolation. Animal husbandry and experimental procedures were performed according to UK Home Office regulations and institute guidelines, under project license 70/8530.

### Cell isolation

Dissected livers (a total of 12) were minced finely with opposing scalpel blades. The tissue was collected in HBSS with Ca ^2+^ Mg ^2+^ (Life Technologies, Paisley, UK) supplemented with 0.01% collagenase IV (Life Technologies) and 0.001% DNase I (Roche, distributed by Sigma-Aldrich, Dorset, UK) and passed through a 70 μm cell strainer. The suspension was spun down (500 × *g*, 4°C, 10 minutes) and the pellet resuspended in RPMI 1640 medium (Life Technologies). The cell suspension was then layered over 24% Optiprep (Sigma-Aldrich) and centrifuged without braking (700 × *g*, RT, 20 minutes). The interface layer was taken and washed in HBSS without Ca ^2+^ Mg ^2+^ (Lonza, distributed by VWR, Lutterworth, UK) supplemented with 0.25% bovine serum albumin (Sigma-Aldrich) and 0.001% DNase I.

Small intestine lamina propria lymphocytes were isolated using a protocol adapted from Halim and Takei ^11^. Briefly, dissected intestines (a total of 12) were placed in ice-cold PBS supplemented with 2% fetal calf serum (FCS; Life Technologies) and the bulk of fecal matter removed by flushing the intestines using a syringe and 18G blunt end needle. The intestines were cut longitudinally and vortexed briefly 3x in ice-cold PBS/2% FCS to remove residual fecal matter. Tissue sections were incubated in PBS supplemented with 1 nM EDTA (shaking at 120 rpm, 37°C, 20 minutes) followed by 3x washes with ice-cold PBS/2% FCS before being minced finely with opposing scalpel blades. The homogenized tissue was digested in DMEM (Life Technologies) supplemented with 10% FCS, 50 µM 2-mercaptoethanol (Life Technologies), 250 U/mL collagenase IV and 50 U/mL DNase I (shaking at 120 rpm, 37°C, 20 minutes) and passed through a 70µm cell strainer. The cell suspension was centrifuged (400 × *g*, 4°C, 5 minutes) and the cell pellet resuspended in 40% Percoll (GE Healthcare, distributed by Sigma-Aldrich) before centrifugation without braking (600 *× g*, 4°C, 10 minutes). The resultant pellet was washed in PBS/2% FCS (400 × *g*, 4°C, 5 minutes).

Dissected spleens (a total of 12) were passed through a 40 µm cell strainer. Red blood cells were lysed by 5 minute incubation in ACK lysing buffer (Life Technologies).

### Flow cytometry

The antibodies used are displayed in Table 1.

**Table 1.** Antibodies used for flow cytometry analysis.

Antibody	Clonality	Fluorophore	Dilution	Host Animal	Manufacturer	Catalog #	RRID
CD3	17A2	FITC	1/200	Rat	Biolegend	100203	AB_312660
CD8α	53-6.7	FITC	1/200	Rat	Biolegend	100705	AB_312744
CD19	6D5	FITC	1/200	Rat	Biolegend	115505	AB_313640
Gr1	RB6-8C5	FITC	1/200	Rat	Biolegend	108405	AB_313370
NK1.1	PK136	APC-eFluor 780	1/200	Mouse	eBioscience	47-5941	AB_2637449
CD45	30-F11	Brilliant Violet 510	1/200	Rat	Biolegend	103137	AB_2561392
CD49a	Ha31/8	Alexa Fluor 647	1/100	Hamster	BD Biosciences	562113	AB_11153312
CD49b	DX5	PerCP-eFluor 710	1/200	Rat	eBioscience	46-5971	AB_11149865
CD127	A7R34	PE	1/100	Rat	eBioscience	17-1271	AB_469435
NKp46	29A1.4	PerCP-eFluor 710	1/50	Rat	eBioscience	46-3351	AB_1834441
Eomes	Dan11mag	PE-eFluor 610	1/100	Rat	eBioscience	61-4875	AB_2574614
Eomes	Dan11mag	PE-Cyanine7	1/100	Rat	eBioscience	25-4875	AB_2573453
Tbet	eBio4B10	eFluor 660	1/100	Mouse	eBioscience	50-5825	AB_10596655
Tbet	eBio4B10	PE-Cyanine7	1/100	Mouse	eBioscience	25-5825	AB_11041809
RORγt	Q31-378	PE-CF594	1/100	Mouse	BD Biosciences	562684	AB_2651150

The lineage cocktail consisted of CD3, CD8α, CD19 and Gr1 (Biolegend, London, UK). Dead cells were excluded using fixable viability dye eFluor 450 (eBioscience, San Diego, CA, USA) (4°C, 15 minutes). Surface staining was carried out in PBS supplemented with 1% FCS (4°C, 15 minutes). Intracellular staining was carried out using Human FoxP3 Buffer (BD Biosciences, Oxford, UK), according to the manufacturer’s instructions. Data were acquired on an LSRFortessa II (BD Biosciences) and analyzed using FlowJo v.X.0.7 (RRID SCR_008520; Tree Star, Ashland, OR, USA).

### Statistical analysis

Groups were compared using Mann-Whitney U Tests. Analysis was carried out using Vassarstats (RRID SCR_010263).

## Results

See Figure 1.

**Figure 1.**
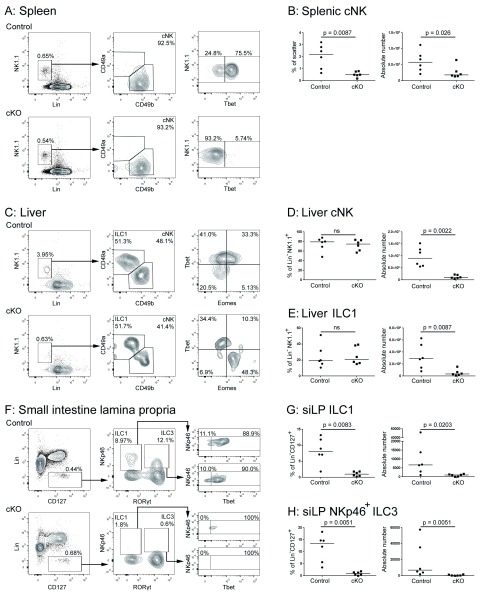
Characterisation of innate lymphoid cell (ILC) populations in Ncr1 ^iCre^ Tbx21 ^fl/fl^ mice. Representative flow cytometry of leukocytes isolated from the ( **A**) spleen, ( **C**) liver and ( **F**) small intestine lamina propria (siLP) of Ncr1 ^WT^ Tbx21 ^fl/fl^ (“control”) and Ncr1 ^iCre^ Tbx21 ^fl/fl^ (“cKO”) mice. Gated by scatter and on live, CD45+ cells. Summary data for cell frequency and absolute number in ( **B**) spleen, ( **D**, **E**) liver and ( **G**, **H**) siLP. Each point represents data from a single mouse (n = 6 per group), bars represent the medians and p values were determined using a Mann-Whitney U Test.

## Discussion

We observed a modest (~4-fold) reduction in splenic NK (defined as Lin- NK1.1+) in the Ncr1 ^iCre^ Tbx21 ^fl/fl^ conditional knockouts, compared to Ncr1 ^WT^ Tbx21 ^fl/fl^ littermate controls ( Figures 1A and B). This is comparable to the 2- to 4-fold reduction in splenic cNK that has previously been reported in Tbx21 knockout, compared to wild-type, mice ^2, 3, 12^ and is likely to be a result of reduced survival ^12^ or bone marrow egress ^13^ of NK cells in the absence of Tbet.

We also observed a reduction in the absolute number of cNK (defined as Lin- NK1.1+ CD49b+) in the liver ( Figures 1C and D). We had expected that this might be similar to the reduction of cNK in the spleen, but, at ~10-fold, it was more pronounced, potentially pointing towards a differential requirement for Tbet in cNK survival in or recruitment to the liver, compared to the spleen. Although the absolute number of liver ILC1 (defined as Lin- NK1.1+ CD49a+) was also reduced (~10-fold), a substantial residual population was present ( Figures 1C and E), in contrast to the Tbx21 knockout, in which these cells are almost completely eliminated ^2, 3^. Unlike the cNK in the conditional knockout, the residual liver ILC1 all expressed Tbet ( Figure 1C). This supports the proposal that Tbet is absolutely required for continued survival of these cells ^2^, since no ILC1 in which Tbet was not expressed persisted. We were surprised to note that Tbx21 excision seemed to be less efficient in liver ILC1 than cNK, because fate mapping of iCre activity under the Ncr1 promoter using R26R ^eYFP^ has previously shown that iCre activity is higher in ILC1 than cNK ^9^. Given the absolute requirement of Tbet for the survival of liver ILC1 ^2, 3^, it seems likely that even if the excision failed in only a few cells, these would have benefited from a selection advantage that might have resulted in their expansion. Whatever the cause of the unexpectedly large reduction in cNK and the unexpectedly small reduction in ILC1, the finding that both of these were reduced by equivalent amounts in the liver of conditional knockouts compared to controls limits the utility of Ncr1 ^iCre^ Tbx21 ^fl/fl^ mice for dissecting the relative contributions of the two cell types in disease models.

Rankin *et al.* have also recently generated Ncr1 ^iCre^ Tbx21 ^fl/fl^ mice, and report a severe reduction in ILC1 (defined in their paper as Lin- RORγt+ NKp46-) and NKp46 ^+^ ILC3 (defined as Lin- RORγt+ NKp46+) in the small intestine lamina propria ^14^. We were able to isolate fewer Lin- CD127+ ILC from the small intestine than has previously been reported, but even with this sub-optimal cell isolation procedure we made findings similar to those of Rankin *et al.*, observing a ~6-fold reduction in ILC1 (defined here as Lin- CD127+ RORγt+ NKp46-) and a ~24-fold reduction in NKp46+ ILC3 (defined here as Lin- CD127+ RORγt+ NKp46+) compared to littermate controls ( Figures 1F–H).

In summary, conditional deletion of Tbet in NKp46 ^+^ cells, where Tbx21 excision has been successful, differentially affects cNK and ILC1 in different organs. In the liver, a residual population of ILC1, in which Tbx21 has not been excised, persists. We conclude that the Ncr1 ^iCre^ Tbx21 ^fl/fl^ mouse is therefore unlikely to be useful for investigating the relative contributions of liver cNK and ILC1 to pathogenesis in disease models, but could still have potential as a tool for understanding how and when Tbet is required for the development and trafficking of NKp46 ^+^ ILC.

## Data availability

The data referenced by this article are under copyright with the following copyright statement: Copyright: © 2017 Cuff AO and Male V

Data is available at DOI, 10.17605/OSF.IO/GDMWT ^10^.
