# Supplementary material for: Conventional NK cells and ILC1 are partially ablated in the livers of Ncr1 iCreTbx21 fl/fl mice
Source: Wellcome Open Res. 2017 Jul 5;2:39. Originally published 2017 Jun 7. [Version 2] doi: 10.12688/wellcomeopenres.11741.2 (PMC5543419; doi:10.12688/wellcomeopenres.11741.2)
Supplement: Peer review history

# Peer review: Referee response for version 2

**DOI:** 10.21956/wellcomeopenres.13074.r24042

**Published:** 2017-07-28

**Authors:** David R. Withers

**Affiliations:** 1 Institute of Immunology and Immunotherapy, Institute of Biomedical Research, University of Birmingham, Birmingham, UK

I have no further comments to make, I approve the manuscript.

I have read this submission. I believe that I have an appropriate level of expertise to confirm that it is of an acceptable scientific standard.

# Peer review: Referee response for version 2

**DOI:** 10.21956/wellcomeopenres.13074.r24041

**Published:** 2017-07-10

**Authors:** Gabrielle T. Belz

**Affiliations:** 1 Walter and Eliza Hall Institute of Medical Research, Melbourne, Vic, Australia

This manuscript is much improved and provides a relevant contribution to the field.

 It would be helpful to extend the discussion on the relevance of this work to already published studies. For example, Pikovskaya et al. (2016) ^1^. This study also indicates that T-bet is inefficiently deleted.

 It remains unclear why NCRiCre controls are not reported in this study as they appear to have been analysed. Nevertheless the authors have added a comment to the rationale of their exclusion from the dataset.

I have read this submission. I believe that I have an appropriate level of expertise to confirm that it is of an acceptable scientific standard.

## References

[1] PikovskayaOChaixJRothmanNJCollinsAChenYHScipioniAMVivierEReinerSL: Cutting Edge: Eomesodermin Is Sufficient To Direct Type 1 Innate Lymphocyte Development into the Conventional NK Lineage. *J Immunol*.2016;196(4) : 10.4049/jimmunol.1502396 1449-54 10.4049/jimmunol.1502396 26792802PMC4744497

# Peer review: Referee response for version 1

**DOI:** 10.21956/wellcomeopenres.12683.r23309

**Published:** 2017-06-20

**Authors:** Gabrielle T. Belz

**Affiliations:** 1 Walter and Eliza Hall Institute of Medical Research, Melbourne, Vic, Australia

This is an interesting small study in which the objective was to delete Tbet and hopefully generate mice that lacked ILC1. This turned out not to be the case.

 This is a very brief report that depends solely on the results shown in Figure 1. These data appear to support the claims of the authors that loss of T-bet is not sufficient to differentially delete ILC1. It is indicated that 6 mice have been used, however, it is not clear whether this represents a single experiment, or alternately, the data are pooled from several experiments. It is essential to clarify this point.

 The authors have used NcrWTTbx21fl/fl control mice for their experiments. Ncr1iCre mice have lower levels of expression of NKp46 compared with either wildtype or floxed control mice. Given only floxed control mice are indicated to have been used, how was this reduction in expression accommodated, or was this considered in the experiments? It is not clear what gating strategy was used for enumerating the NK cell frequency or number.

I have read this submission. I believe that I have an appropriate level of expertise to confirm that it is of an acceptable scientific standard, however I have significant reservations, as outlined above.

# Response

**Published:** 2017-07-03

**Authors:** Victoria Male

**Affiliations:** Royal Free and UCL School of Medicine, UK

This is an interesting small study in which the objective was to delete Tbet and hopefully generate mice that lacked ILC1. This turned out not to be the case.

 This is a very brief report that depends solely on the results shown in Figure 1. These data appear to support the claims of the authors that loss of T-bet is not sufficient to differentially delete ILC1. It is indicated that 6 mice have been used, however, it is not clear whether this represents a single experiment, or alternately, the data are pooled from several experiments. It is essential to clarify this point.

**Six littermate pairs (a total of 12 mice) were used, with spleen, liver and small intestine taken from each mouse. We have clarified this point in the Methods of version 2:**

****

**“Ncr1 ^iCre^ mice were crossed onto Tbx21 ^fl/fl^ and the resultant F1 generation was backcrossed onto Tbx21 ^fl/fl^ to produce Ncr1 ^iCre^ Tbx21 ^fl/fl^ conditional knockouts (n = 6) and Ncr1 ^WT^ Tbx21 ^fl/fl^ littermate controls (n = 6)… Spleen, liver and intestines were dissected out of each of the 12 mice for cell isolation.”**

 The authors have used NcrWTTbx21fl/fl control mice for their experiments. Ncr1iCre mice have lower levels of expression of NKp46 compared with either wildtype or floxed control mice. Given only floxed control mice are indicated to have been used, how was this reduction in expression accommodated, or was this considered in the experiments? It is not clear what gating strategy was used for enumerating the NK cell frequency or number.

**In version 2, we discuss the reported phenotype of Ncr1 ^iCre^ mice and the steps we have taken to avoid the potential confounding effect of slightly lower NKp46 expression in NKp46+ cells in these mice; in particular, that we identify NK cells using NK1.1, rather than NKp46. We have also made the data from our own characterisation of Ncr1 ^iCre^ compared to WT littermate control mice available, linked to this publication.**

****

**“Although we chose to use floxed-only, rather than iCre-only, littermate controls, we do recognise that iCre transgene expression itself can have an effect on phenotype. Ncr1 ^iCre^ mice are known to have slightly reduced expression of NKp46 on NK cells, although the total number of NK cells (identified as CD3- NKp46+) in these mice is normal ^9^. We confirmed these observations in Ncr1 ^iCre^ mice, compared to Ncr1 ^WT^ littermate controls in our own colony ^15^. Further, we identify NK cells as Lin- NK1.1+, rather than Lin- NKp46+, to avoid potential confounding effects of reduced NKp46 expression.”**

****

**Gating strategies are as shown in the figure. For greater clarity, in version 2 we also give the gating strategies used to define each population in the text.**

# Peer review: Referee response for version 1

**DOI:** 10.21956/wellcomeopenres.12683.r23310

**Published:** 2017-06-08

**Authors:** David R. Withers

**Affiliations:** 1 Institute of Immunology and Immunotherapy, Institute of Biomedical Research, University of Birmingham, Birmingham, UK

The manuscript of Cuff and Male provides useful insight into the effects of deleting *tbx21* in cells expressing NK1.1, resulting in loss of T-bet expression in a subset of immune cells. In the current climate of developing our understanding of mechanisms using conditional KO mice, basic data on how models work has clear value to the immunological community and data indicating the success of problems with this type of model can really benefit other researchers. It is also important for researchers to fully assess how well different conditional KO mouse models work with robust controls – it is now apparent that one cannot simply assume either specific or efficient cre-mediated deletion simply because of the intentioned design of the mouse model. Within the data presented here, it is striking that tissue specific effects are observed, particularly given the interest in tissue residency of ILC populations and the potential for tissue-specific differentiation. Much of the data is clearly presented and appears robust in terms of the analysis of NK/ILC1 populations. There are some modest concerns with manuscript as it stands - some in terms of presentation of the data and some in terms of the experimental setup and methodology.

 Experimental design:

- The control mice used here are ‘floxed only’ controls rather than ‘cre only’ controls. The latter is better as it enables the impact of expression of the bacteriophage protein (cre), which is clearly a ‘foreign’ element expressed in the mouse. A number of studies have demonstrated that cre expression can impact on aspects of the immune system (e.g. Lck in T cell development in the thymus). This should be acknowledged within the description of the data.
- The authors should have fate-mapped cre expression in their mice in their hands to enable the efficiency of cre-expression to be properly addressed. This would clarify the extent to which NK1.1 expressing cells show evidence of cre mediated deletion, informing the understanding of NK1.1+ cells that retain T-bet expression. Whilst beyond the scope of this study now, it again could be noted.

Methodology:

- The SI LP prep is clearly sub-optimal for ILC populations – the fraction of the Lineage negative cells expressing IL-7Rα is substantially less than seen with other methods and there is clearly a substantial range in terms of numbers of cells isolated. Thus the data from the SI LP prep is of limited value given the spread of data and the obvious issues in cell isolation. Thus this data appears very preliminary and suboptimal - this should be recognised in the description of this data.

Presentation of data:

- In Figures B,D, E, G and H it would be more appropriate to show the median rather than the mean (which is what I assume the bar represents – it is not described in the figure legend). One cannot assume the data is normally distributed and plotting the median would eliminate bias driven by clear outliers - for example the data in Figure H, absolute number clearly suffers from this, as does the similar data in ‘G’. The data in all these graphs should show median, not mean and this should be articulated in the Figure Legend.
- It is not clear to me in the SI LP analysis (in part ‘F’) why Lin-IL-7Ra+ cells are not gated on – there is a clear IL-7Ra+ population, but the gate includes IL-7Ra- cells. Given that the aim of this analysis is to look at ILC populations (but with no reference to conventional NK cells which are IL-7Ra-), a more specific gate would seem sensible – this would be my recommendation.
- Also in part F, the plots of T-bet expression would benefit from an indication of where the authors consider +ve and –ve T-bet staining. This would seem reasonably uncontentious given the populations observed.

I have read this submission. I believe that I have an appropriate level of expertise to confirm that it is of an acceptable scientific standard, however I have significant reservations, as outlined above.

# Response

**Published:** 2017-07-03

**Authors:** Victoria Male

**Affiliations:** Royal Free and UCL School of Medicine, UK

The control mice used here are ‘floxed only’ controls rather than ‘cre only’ controls. The latter is better as it enables the impact of expression of the bacteriophage protein (cre), which is clearly a ‘foreign’ element expressed in the mouse. A number of studies have demonstrated that cre expression can impact on aspects of the immune system (e.g. Lck in T cell development in the thymus). This should be acknowledged within the description of the data.

**In version 2, we acknowledge that Cre expression itself can have a phenotypic effect. We discuss the reported phenotype of Ncr1 ^iCre^ mice and the steps we have taken in this study to avoid the potential confounding effect of slightly lower NKp46 expression in NKp46+ cells in these mice, in particular that we identify NK cells using NK1.1, rather than NKp46. We have also made the data from our own characterisation of Ncr1 ^iCre^ compared to WT littermate control mice available, linked to this publication. In “Mice”:**

****

**“Although we chose to use floxed-only, rather than iCre-only, littermate controls, we do recognise that iCre transgene expression itself can have an effect on phenotype. Ncr1 ^iCre^ mice are known to have slightly reduced expression of NKp46 on NK cells, although the total number of NK cells (identified as CD3- NKp46+) in these mice is normal ^9^. We confirmed these observations in Ncr1 ^iCre^ mice, compared to Ncr1 ^WT^ littermate controls in our own colony ^15^. Further, we identify NK cells as Lin- NK1.1+, rather than Lin- NKp46+, to avoid potential confounding effects of reduced NKp46 expression.”**

 The authors should have fate-mapped cre expression in their mice in their hands to enable the efficiency of cre-expression to be properly addressed. This would clarify the extent to which NK1.1 expressing cells show evidence of cre mediated deletion, informing the understanding of NK1.1+ cells that retain T-bet expression. Whilst beyond the scope of this study now, it again could be noted.

**Although we have not done this ourselves, fate mapping of the Ncr1 ^iCre^ mice was done as part of the characterisation when they were first developed. We have added this to the Discussion of version 2:**

****

**“We were surprised to note that Tbx21 excision seemed to be less efficient in liver ILC1 than cNK, because fate mapping of iCre activity under the Ncr1 promoter using R26R ^eYFP^ has previously shown that iCre activity is higher in ILC1 than cNK ^9^.”**

 The SI LP prep is clearly sub-optimal for ILC populations – the fraction of the Lineage negative cells expressing IL-7Rα is substantially less than seen with other methods and there is clearly a substantial range in terms of numbers of cells isolated. Thus the data from the SI LP prep is of limited value given the spread of data and the obvious issues in cell isolation. Thus this data appears very preliminary and suboptimal - this should be recognised in the description of this data.

**We acknowledge this in version 2. In the Discussion:**

****

**“We were able to isolate fewer Lin- CD127+ ILC from the small intestine than has previously been reported, but even with this sub-optimal cell isolation procedure we made findings similar to that of Rankin *et al.*, observing a ~6-fold reduction in ILC1 (defined here as Lin- CD127+ RORγt+ NKp46-) and a ~24-fold reduction in NKp46+ ILC3 (defined here as Lin- CD127+ RORγt+ NKp46+) compared to littermate controls ( Figures 1F–H).”**

 In Figures B,D, E, G and H it would be more appropriate to show the median rather than the mean (which is what I assume the bar represents – it is not described in the figure legend). One cannot assume the data is normally distributed and plotting the median would eliminate bias driven by clear outliers - for example the data in Figure H, absolute number clearly suffers from this, as does the similar data in ‘G’. The data in all these graphs should show median, not mean and this should be articulated in the Figure Legend.

**We have done this in version 2.**

 It is not clear to me in the SI LP analysis (in part ‘F’) why Lin-IL-7Ra+ cells are not gated on – there is a clear IL-7Ra+ population, but the gate includes IL-7Ra- cells. Given that the aim of this analysis is to look at ILC populations (but with no reference to conventional NK cells which are IL-7Ra-), a more specific gate would seem sensible – this would be my recommendation.

**Because our aim in the siLP analysis was to confirm the findings from Rankin et al, we used their gating strategy: ILC1 and NKp46+ ILC3 as a proportion of total lineage negative cells. However, we do agree that gating on Lin- CD127+ cells is more appropriate for identifying ILCs and we have reanalysed the data using this strategy in version 2. Readers who wish to compare our data directly to that of Rankin et al may refer to version 1, which will still be available.**
